# Profilin-1 Is Expressed in Human Atherosclerotic Plaques and Induces Atherogenic Effects on Vascular Smooth Muscle Cells

**DOI:** 10.1371/journal.pone.0013608

**Published:** 2010-10-25

**Authors:** Evren Caglayan, Giulio R. Romeo, Kai Kappert, Margarete Odenthal, Michael Südkamp, Simon C. Body, Stanton K. Shernan, Daniel Hackbusch, Marius Vantler, Andrius Kazlauskas, Stephan Rosenkranz

**Affiliations:** 1 Klinik III für Innere Medizin, Universität zu Köln, Cologne, Germany; 2 Center for Molecular Medicine Cologne (CMMC), Universität zu Köln, Cologne, Germany; 3 Department of Cellular and Molecular Physiology, Joslin Diabetes Center, Boston, Massachusetts, United States of America; 4 Institut für Pharmakologie, Center for Cardiovascular Research (CCR), Charité-Universitätsmedizin Berlin, Berlin, Germany; 5 Institut für Pathologie, Universität zu Köln, Cologne, Germany; 6 Herz- und Gefäßchirurgie, Universitätsklinikum Freiburg, Freiburg, Germany; 7 Brigham and Women's Hospital, Harvard Medical School, Boston, Massachusetts, United States of America; 8 Schepens Eye Research Institute, Harvard Medical School, Boston, Massachusetts, United States of America; University of Tor Vergata, Italy

## Abstract

**Background:**

Profilin-1 is an ubiquitous actin binding protein. Under pathological conditions such as diabetes, profilin-1 levels are increased in the vascular endothelium. We recently demonstrated that profilin-1 overexpression triggers indicators of endothelial dysfunction downstream of LDL signaling, and that attenuated expression of profilin-1 confers protection from atherosclerosis *in vivo*.

**Methodology:**

Here we monitored profilin-1 expression in human atherosclerotic plaques by immunofluorescent staining. The effects of recombinant profilin-1 on atherogenic signaling pathways and cellular responses such as DNA synthesis (BrdU-incorporation) and chemotaxis (modified Boyden-chamber) were evaluated in cultured rat aortic and human coronary vascular smooth muscle cells (VSMCs). Furthermore, the correlation between profilin-1 serum levels and the degree of atherosclerosis was assessed in humans.

**Principal Findings:**

In coronary arteries from patients with coronary heart disease, we found markedly enhanced profilin expression in atherosclerotic plaques compared to the normal vessel wall. Stimulation of rat aortic and human coronary VSMCs with recombinant profilin-1 (10^−6^ M) *in vitro* led to activation of intracellular signaling cascades such as phosphorylation of Erk1/2, p70^S6^ kinase and PI3K/Akt within 10 minutes. Furthermore, profilin-1 concentration-dependently induced DNA-synthesis and migration of both rat and human VSMCs, respectively. Inhibition of PI3K (Wortmannin, LY294002) or Src-family kinases (SU6656, PP2), but not PLCγ (U73122), completely abolished profilin-induced cell cycle progression, whereas PI3K inhibition partially reduced the chemotactic response. Finally, we found that profilin-1 serum levels were significantly elevated in patients with severe atherosclerosis in humans (*p*<0.001 vs. no atherosclerosis or control group).

**Conclusions:**

Profilin-1 expression is significantly enhanced in human atherosclerotic plaques compared to the normal vessel wall, and the serum levels of profilin-1 correlate with the degree of atherosclerosis in humans. The atherogenic effects exerted by profilin-1 on VSMCs suggest an auto-/paracrine role within the plaque. These data indicate that profilin-1 might critically contribute to atherogenesis and may represent a novel therapeutic target.

## Introduction

Diabetes and dyslipidemia are major risk factors for atherosclerotic vascular disease. It remains however unclear how these entities contribute to atherogenesis. Although diabetes is associated with a marked accumulation of vascular smooth muscle cells (VSMC) in fibroatheromas, representing an integral part of atherosclerotic lesion progression, high glucose and insulin levels do not directly induce VSMC proliferation and accumulation *in vivo* [1]. It has been proposed that suppression of insulin signaling in insulin resistance interferes with protective actions of insulin such as endothelial NO production and anti-inflammatory effects and thus leads to a chronic state of (pro)inflammation [2], [3], representing a hallmark of atherosclerosis [4]. Hyperglycemia can induce endothelial dysfunction and attenuates vascular endothelial growth factor (VEGF) signaling in endothelial cells (ECs), thereby impairing the regenerative capacity of the endothelium [3], [5]. Finally, the pro-insulin cleavage product C-peptide may also contribute to vascular dysfunction, as it co-localizes with monocytes in early atherosclerotic lesions in type 2 diabetics, and serves as a chemoattractant for human monocytes and a mitogen for VSMCs [6], [7]. While some mediators have thus been identified, the exact cellular mechanisms that lead to accelerated atherosclerosis remain poorly understood.

By screening of a peptide phage display library, we previously identified profilin-1 as a binding partner for a diabetic aorta-specific phage in rats [8]. Profilin-1 was increased in the endothelium of diabetic animals, and its overexpression in cultured ECs triggered indicators of endothelial dysfunction (apoptosis, ICAM-1 expression, decreased NO signaling) [8]. More recently, we specified the importance of profilin-1 for atherogenesis *in vivo*, as profilin-1 heterozygosity conferred protection from atherosclerosis in LDL receptor-null mice [9].

Profilin-1 is commonly recognized as an intracellular actin-binding protein [10], which regulates the size, localization and dynamics of unpolymerized actin in cells, and thus contributes to cytoskeletal dynamics and actin-dependent cellular processes [11]. Intracellular profilin-1 interacts with many ligands such as actin, the actin-related protein (Arp)2, gephyrin, phosphatidyl-inositol-4,5-bisphosphate (PIP2), and diverse proteins comprising a poly-proline stretch, i.e. VASP, p140mDia, and neuronal Wiscott-Aldrich-Syndrome protein [12]. Through these interactions, profilin-1 also serves as a signaling protein, transmitting external signals from the cell surface to microfilaments [13]. However, since there is also evidence for a pathogenic role of extracellular profilin-1 in diseases states such as glomerulonephritis and after endothelial damage [14], [15], we now investigated whether profilin-1 is present in human atherosclerotic plaques, and whether it induces atherogenic responses in VSMCs.

## Methods

### Human tissue samples and immunofluorescence staining

Coronary arteries were collected from 8 adults with severe coronary artery disease. All patients had an unfavorable risk profile including diabetes/impaired glucose tolerance, hypertension, and dyslipidemia. Collections were approved by the local Ethics Committee of the University of Cologne, and informed consent was obtained from each patient. Segments of diseased or undiseased regions were sampled and fixed in 4% formaldehyde solution. Serial transverse sections were processed, and stained with Elastin van Gieson (EvG) and Masson's trichrome. Immunofluorescence stainings were performed by incubating deparaffinized sections with a rabbit anti-profilin-1 polyclonal antibody (1∶50), a monoclonal mouse anti α-smooth-muscle actin (SMA) antibody (DAKO, Clone 1A4; 1∶100) or a monoclonal mouse anti von-Willebrand Factor (vWF) antibody (1∶100). Profilin-1, SMA and vWF were visualized by a Cy3-conjugated (Jackson Immunoresearch, West Grove, PA, USA) and an Alexa Fluor® 488-conjugated secondary antibody (Molecular Probes, donkey anti–mouse IgG), respectively. Nuclei were counterstained with DAPI. Non-immune rabbit IgG was used as a negative control in consecutive sections.

### Synthesis and Purification of recombinant rat profilin and human profilin

Full-length rat profilin subcloned into pQE-30 (Qiagen, Valencia, CA, USA) downstream of a 6His-tag (kindly provided by M. Tamura) was expressed in M15 *E. coli* as previously described [8]. Profilin was purified under native conditions by Ni^++^ affinity chromatography using Ni-NTA agarose (Qiagen, Valencia, CA, USA) according to the manufacturers instructions. Human recombinant profilin-1 was purchased from Abcam. Endotoxin levels in the profilin preparation were below the detection threshold (<0.1 ng/µg) as assessed by the limulus assay.

### Cell Culture and cellular responses

Rat VSMCs were isolated from thoracic aorta (Wistar Kyoto; 6–10 wk old; Charles River Wega GmbH, Sulzfeld, Germany) by enzymatic dispersion and cultured as previously described [16], [17], [18]. Human coronary VSMCs were purchased from Lonza Biosciences (Vervier, Belgium) and maintained as recommended by the manufacturer. Profilin-dependent DNA synthesis was measured by a 5-bromodeoxyuridine (BrdU)-incorporation assay as described [18]. Cells were synchronized by serum-deprivation for at least 24 hours and stimulated with various concentrations of recombinant profilin (1 nM–10 µM) for 24 hours. BrdU incorporation was measured after an incubation time of 16 hours. Chemotaxis was assayed using a 48-well modified Boyden chamber (NeuroProbe, Baltimore, MD) and PVP-free polycarbonate filters (8-µm pores) (Poretics, Livermore, CA) as described [17]. Synchronized VSMCs were allowed to migrate for 5 hours at 37°C. Pharmacological inhibitors against PI 3-kinase (Wortmannin, LY294002), MEK (PD98059), Src family kinases (SU6656; PP2), and PLCγ (U73122) were purchased from Calbiochem.

### Phosphorylation of signaling molecules

Subconfluent VSMCs were growth-arrested by serum deprivation for 24 h and subsequently stimulated with recombinant profilin-1 (up to 1 µM) for various time points in the presence or absence of pharmacological inhibitors as indicated. The cells were harvested, the lysates were resolved by SDS-PAGE, and subjected to Western blotting as described [17], [18], using antisera against RasGAP (lysate control), phospho-Erk1/2 (thr202/tyr204), phospho-Akt (ser473), or phospho-p70^S6K^ (Cell Signaling). The Western blots were semi-quantitatively analyzed by densitometry, and all data were normalized for RasGAP.

### Quantitative real-time PCR

Profilin transcripts were measured ex vivo in carotid arteries from 6-month-old male LDLR-deficient mice that were exposed to chow or atherogenic diet for 3 months. RNA was isolated and quantitatve real-time PCR was performed using SYBR green as previously published [19]. Primer pairs were as follows: Profilin forward (5′-3′): TTACGCCAGCTGAGGTTGGT; profilin reverse (5′-3′): CCACCGTGGACACCTTCTTT; 18S forward (5′-3′): GGACTCTTTCGAGGCCCTGTA; 18S reverse (5′-3′): CACCAGACTTGCCCTCCAAT. All animal experiments have been performed according to national and international guidelines. The protocol for the animal experiments was submitted to, and approved by, the local animal research authority.

### Quantification of atherosclerosis in humans

104 patients undergoing coronary artery bypass grafting (CABG) were stratified for aortic atherosclerosis, evaluated at 33 points along the arch, thoracic and abdominal aorta using intraoperative ultrasound. Depending on the degree of aortic atherosclerosis, individuals were clustered in three categories according to the 33-point ultrasound score (0–7 = None; 8–20 = Mixed; 21–33 = Severe). 18 individuals without major diagnosed diseases served as a control group.

### Semi-quantitative analysis of profilin-1 serum levels

Profilin was immunoprecipitated from 400 µl of serum from each patient (Santa Cruz; sc-166191), and Western blot analysis was performed using an antibody that recognizes human profilin-1 (Cell Signaling; Ab3237). Semi-quantitative analysis was performed using a series of dilution of recombinant profilin in the same gel.

### Statistical analysis

All data are expressed as means ± SEM. Statistical analysis was performed using paired or unpaired *t* test or one way analysis of variance (ANOVA) followed by Newman-Keuls post-hoc test for multiple comparisons as appropriate. *P*<0.05 was considered statistically significant.

## Results

### Profilin-1 is expressed in human coronary atherosclerotic plaques

**Figure 1:**
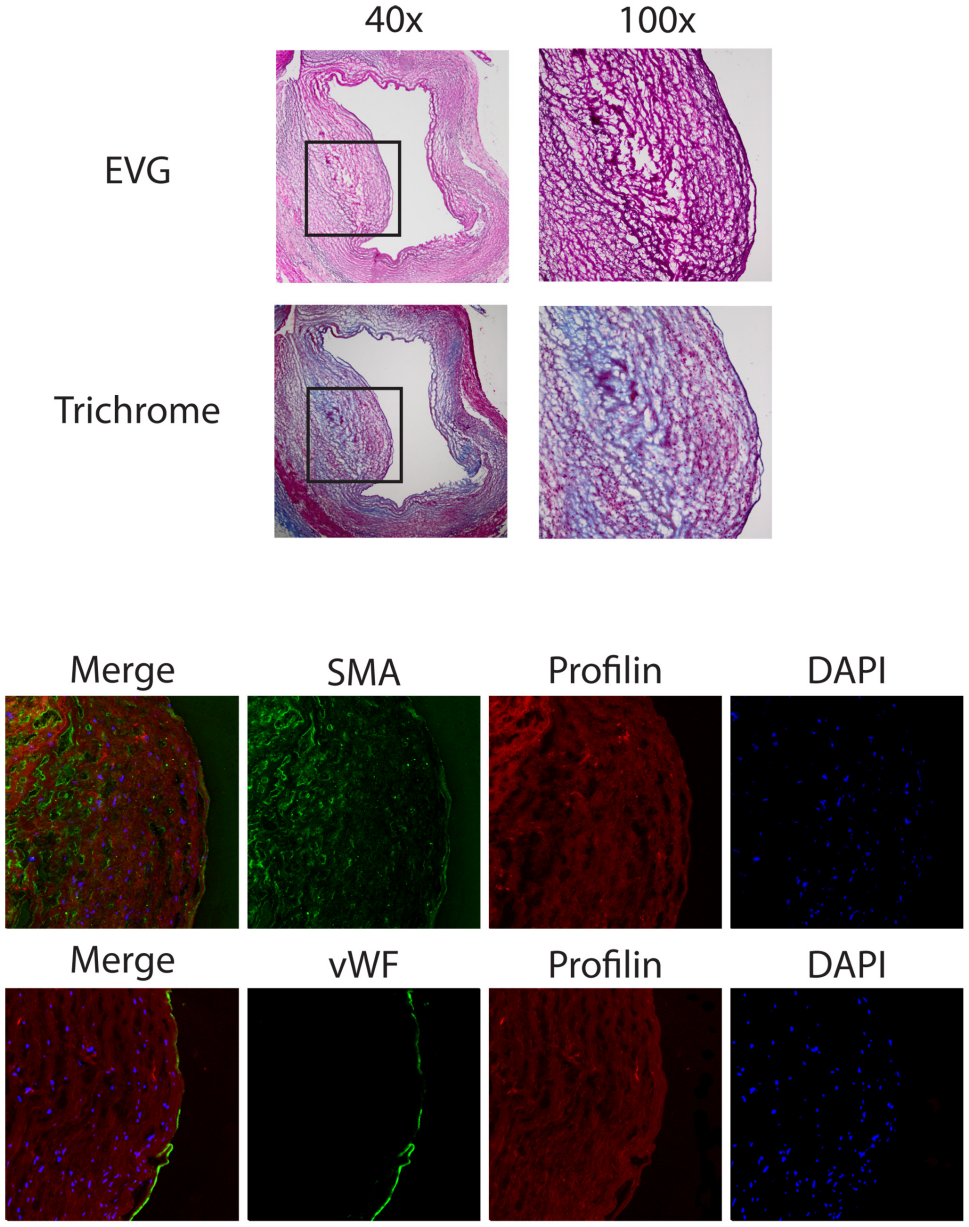
Expression of profilin in human atherosclerotic plaque. Analysis of consecutive sections from a coronary artery of a representative patient with coronary artery disease. *Upper panel*: EvG and Masson's trichrome staining at 40×, inset was magnified at 100×. *Middle and lower panels*: Immunofluorescence staining for profilin (red), α-smooth muscle actin (SMA, green), and von Willebrand Factor (vWF, green). DAPI-staining (blue) was performed to visualize nuclei.

**Figure 2:**
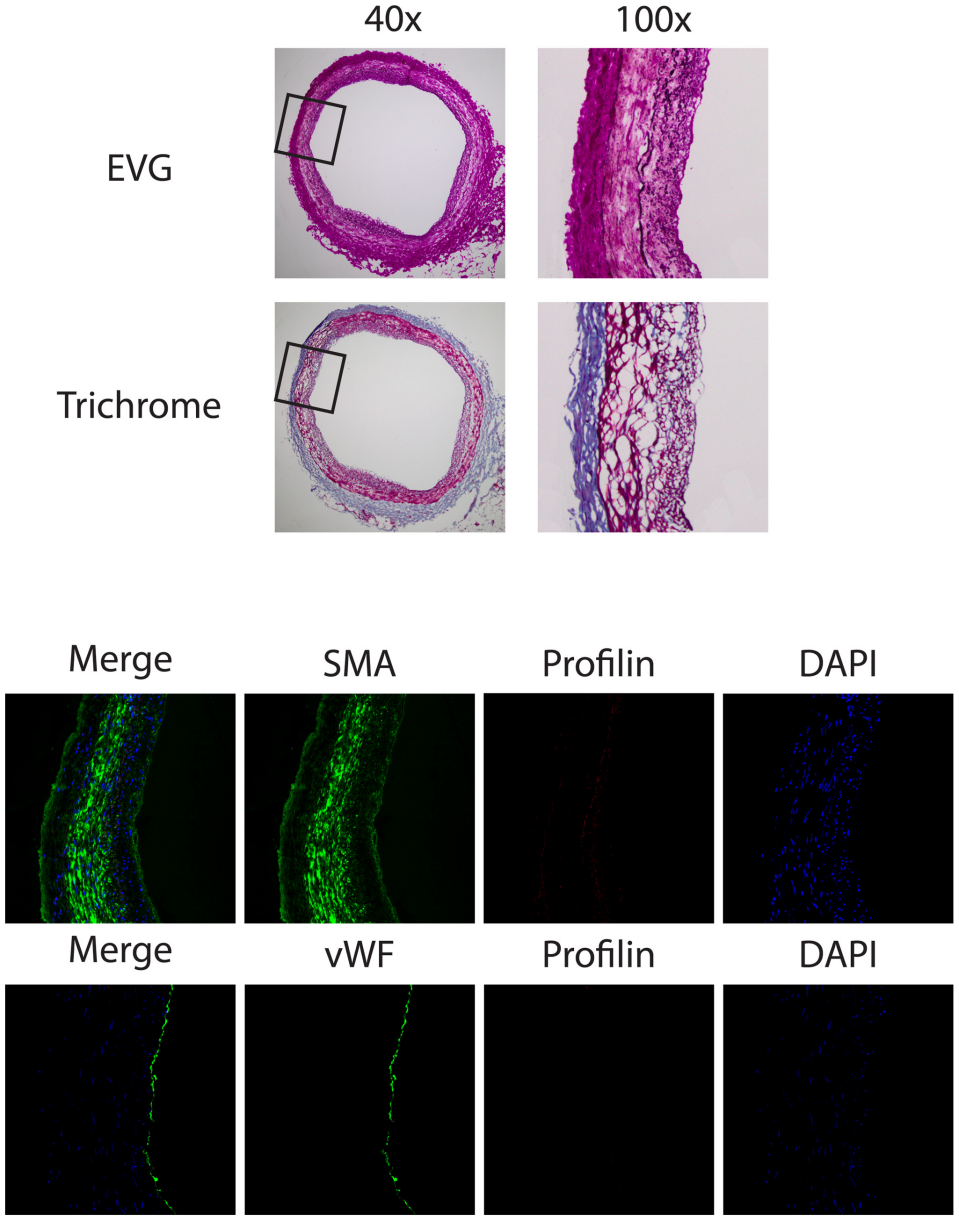
Low expression of profilin in normal coronary vessel. Analysis of consecutive sections from a representative coronary artery without evident coronary artery disease. *Upper panel*: EvG and Masson's trichrome staining at 40×, inset was magnified at 100×. *Middle and lower panels*: Immunofluorescence staining for profilin (red), α-smooth muscle actin (SMA, green), and von Willebrand Factor (vWF, green). DAPI-staining (blue) was performed to visualize nuclei.

Coronary arteries were obtained from 8 patients with coronary artery disease who underwent heart transplantation. Profilin-1 expression was highly abundant within atherosclerotic lesions when compared to the adjacent tissue (**Fig. 1**). Moreover, co-staining for VSMCs (SMA) or endothelial cells (vWF) revealed that profilin-1 expression was not restricted to the endothelium, and profilin-1 was also present in the extracellular space. Negative controls were performed using non-immune IgG to rule out unspecific binding of the profilin antibody (data not shown). Similar findings were observed in all patients. In contrast to the abundant expression in atherosclerotic plaques, profilin-1 was not expressed at significant levels in non-atherosclerotic coronary arteries (**Fig. 2**).

### Profilin-1 induces cellular responses and activates classical signaling cascades in rat and human vascular smooth muscle cells

To further evaluate whether extracellular profilin may have a functional role within atherosclerotic plaques, we investigated its ability to directly initiate cellular responses relevant to atherogenesis in VSMCs, such as cell cycle progression and migration via activation of classical signaling cascades.

**Figure 3:**
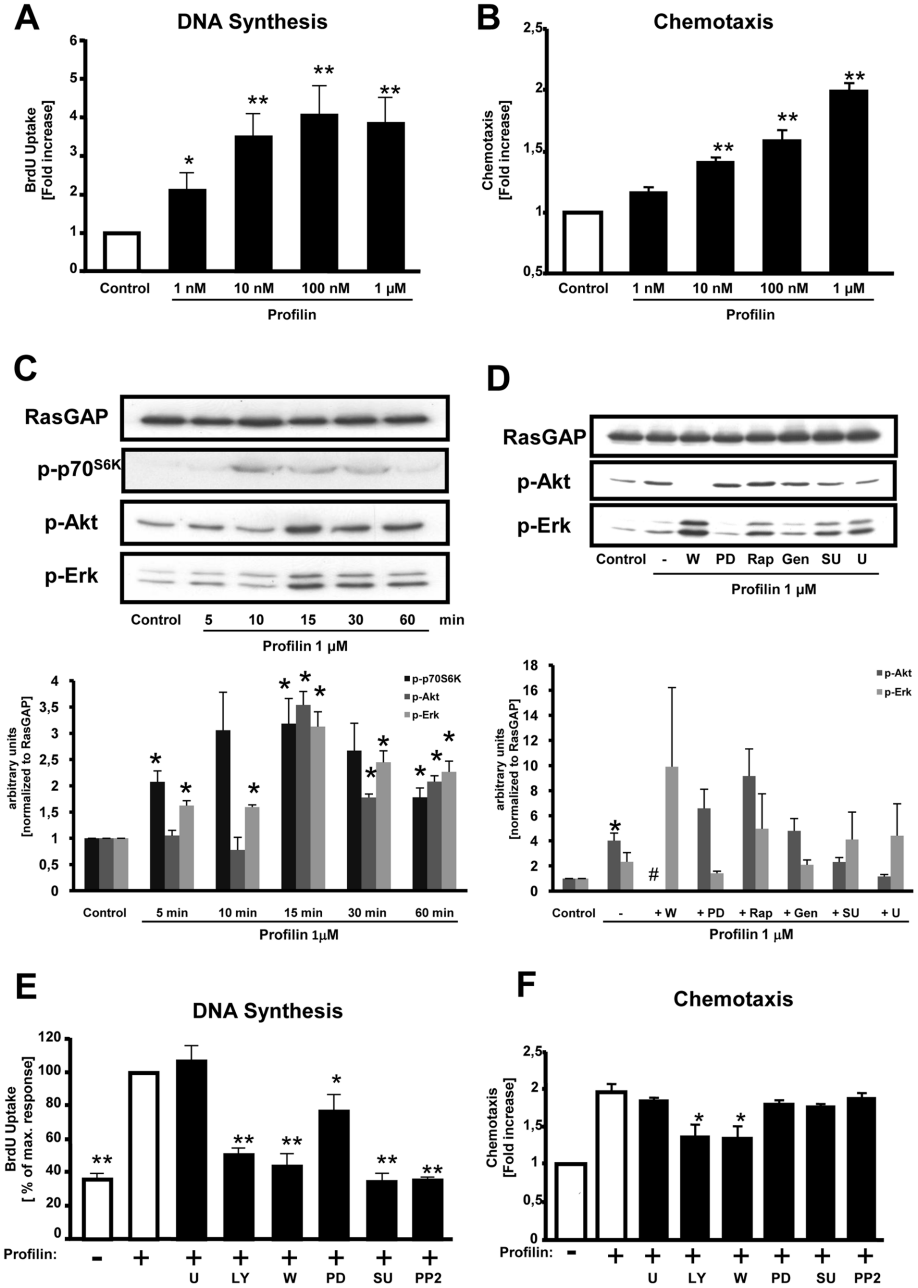
Profilin induces cellular responses and activates classical signaling cascades in rat VSMCs. (**A**) DNA-synthesis as assessed by measurement of BrdU incorporation. (**B**) Chemotaxis was evaluated utilizing modified Boyden chemotaxis chambers. (**C**) Quiescent VSMCs were stimulated with recombinant profilin-1 (1 µM) for various time points as indicated. The cells were lysed, and equal amounts of protein were subjected to SDS-PAGE and Western blot analyses, using phospho-specific antibodies recognizing phosphorylated p70^S6K^, Akt, and Erk1/2. RasGAP served as a lysate control. (**D**) Cells were stimulated with recombinant profilin-1 (1 µM) for 15 min in the presence of pharmacological inhibitors as indicated. Data in C and D were quantified by densitometry and are expressed as fold increase compared to buffer-treated control cells. (**E**) DNA synthesis was assessed by measurement of BrdU incorporation, and recombinant profilin was added in the presence of pharmacological inhibitors as indicated. Data are expressed as the percentage of the maximal profilin-response. (**F**) Chemotaxis was evaluated utilizing modified Boyden chemotaxis chambers in the presence of pharmacological inhibitors. Data are expressed as fold increase compared to buffer-treated control cells. Pharmacological inhibitors: PI3K inhibitors wortmannin (W; 100 nM) and LY294002 (LY; 20 µM), MEK inhibitor PD98059 (PD; 30 µM), rapamycin (Rap; 10 nM), tyrosine kinase inhibitor genestein (Gen; 50 µM), Src inhibitors SU6656 (SU; 2.5 µM) or PP2 (50 nM), PLCγ inhibitor U73122 (U; 10 µM). All data represent means ± SEM from at least three independent experiments. *P<0.05, **P<0.01 vs. control.

**Figure 4:**
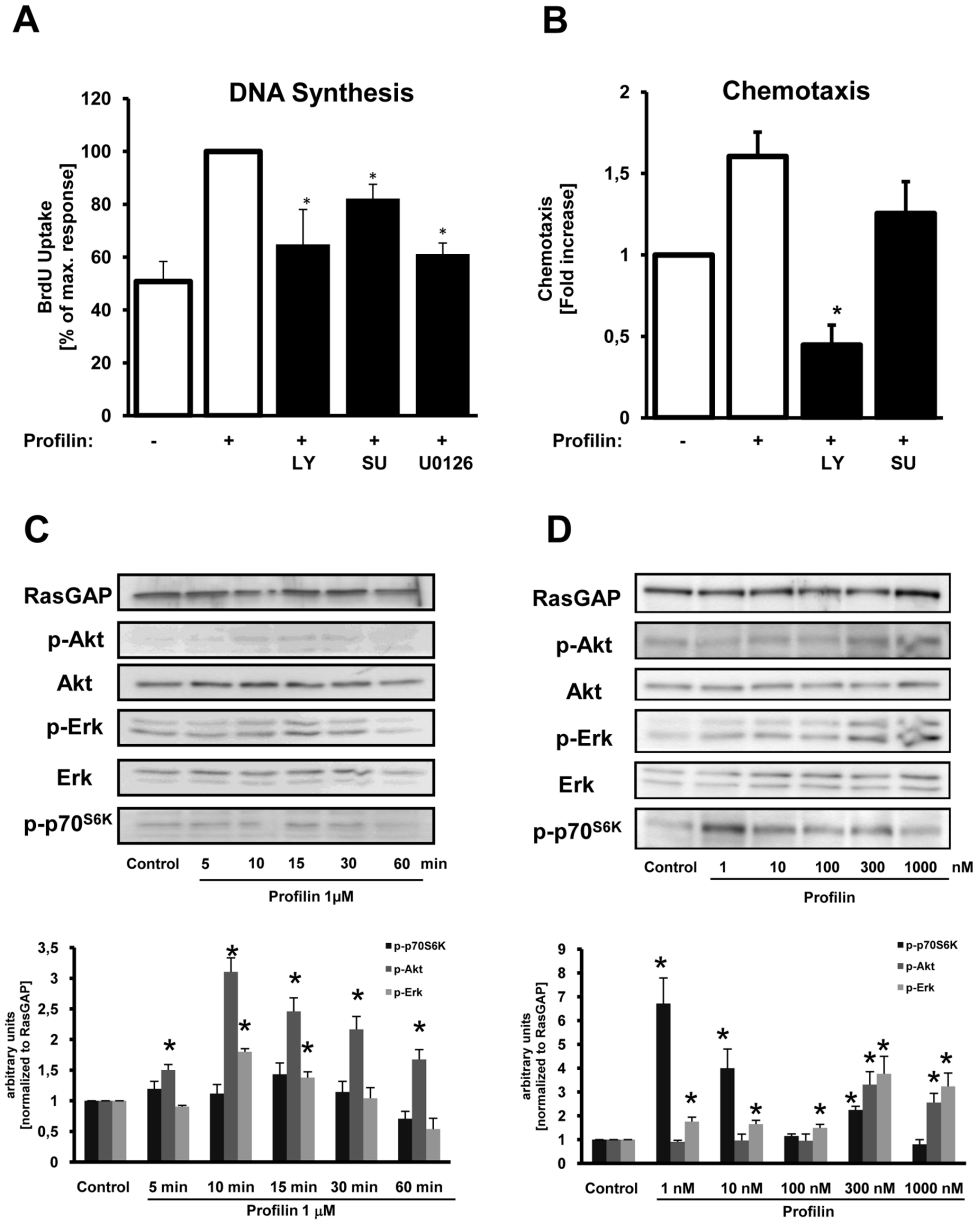
Profilin induces cellular responses and activates classical signaling cascades in human coronary VSMCs. (**A**) DNA-synthesis as assessed by measurement of BrdU incorporation in the absence or presence of inhibitors as indicated. Data are expressed as the percentage of the maximal profilin-response. (**B**) Chemotaxis was evaluated utilizing modified Boyden chemotaxis chambers in the absence or presence of inhibitors as indicated. Data are expressed as fold increase compared to buffer-treated control cells. See Figure 3 for inhibitors. Data in A and B represent means ± SEM from at least three independent experiments. *P<0.05, **P<0.01 vs. control. (**C and D**) Quiescent VSMCs were stimulated with recombinant profilin-1 (1 µM) for various time points and at various concentrations as indicated. The cells were lysed, and equal amounts of protein were subjected to SDS-PAGE and Western blot analyses, using phospho-specific antibodies recognizing phosphorylated p70^S6K^, Akt, and Erk1/2. RasGAP served as a lysate control. Data in C and D were quantified by densitometry and are expressed as fold increase compared to buffer-treated control cells.

Stimulation of quiescent rat VSMCs with recombinant profilin-1 led to a concentration-dependent increase of DNA synthesis to maximally 3.9±0.6-fold at 1 µM compared to non-stimulated cells (*P*<0.05) (**Fig. 3A**). Likewise, profilin-1 at 1 µM also induced DNA synthesis 2.7±0.7-fold in human coronary VSMCs (*P*<0.05) (**Fig. 4A**). Profilin-1 also dose-dependently induced the migration of rat VSMCs to maximally 2.0±0.1-fold (*P*<0.01) (**Fig. 3B**) as well as the migration of human coronary VSMCs to 1.6±0.1-fold (*P*<0.01) at 1 µM (**Fig. 4B**).

Growth factors induce cell cycle progression and chemotaxis in various cell types via activation of classical signaling pathways such as the PI 3-kinase (PI3K)/Akt (PKB) pathway, activation of Src family kinases, phospholipase-Cγ1 (PLCγ), or the Ras-Raf-MEK-Erk pathway, which ultimately leads to the phosphorylation/activation of extracellular-regulated kinase 1/2 (Erk1/2). Stimulation of VSMCs with recombinant profilin-1 (1 µM) led to a rapid and sustained phosphorylation of Erk 1/2, Akt, and p70^S6K^ within 15 minutes in both rat and human cells that was time- and concentration-dependent (**Fig. 3C** **and** **4C** **/D**). Profilin-induced Erk phosphorylation was potently inhibited by the MEK1 inhibitor PD98059, whereas profilin-dependent Akt phosphorylation was inhibited by the PI3K inhibitors Wortmannin and LY294002 (**Fig. 3D**), indicating that these downstream targets are activated by profilin-1 via the classical Ras-Raf-MEK- and PI3K-dependent pathways. It is interesting to note, that inhibition of PI3K with Wortmannin or LY294002 potentiated Erk phosphorylation. As expected, inhibitors of PLCγ (U73122) or Src family kinases (SU6656, PP2) did not affect Akt and Erk phosphorylation, whereas the tyrosine kinase inhibitor genistein (Gen) slightly inhibited profilin-dependent Erk activation.

### Identification of intracellular signaling pathways that mediate profilin-dependent cellular responses

To better understand the molecular mechanisms that mediate profilin-induced DNA synthesis and migration in VSMCs, we investigated these cellular responses in the presence of pharmacological inhibitors. Profilin-dependent cell cycle progression in rat aortic VSMCs was almost completely abolished by the PI3K inhibitors Wortmannin and LY294002 as well as the Src inhibitors SU6656 and PP2, whereas inhibition of PLCγ with U73122 did not affect this cellular response (**Fig. 3E**). Inhibition of the Erk pathway by PD98059 reduced BrdU uptake upon profilin-1 stimulation by approximately 50%. Similar results were obtained in human coronary VSMCs (**Fig. 4A**). These data indicate that profilin-dependent DNA synthesis requires PI3K, Src, and, to a lesser extent, Erk1/2. The profilin-induced migration of VSMCs was reduced by inhibition of PI3K (Wortmannin, LY294002), whereas all other inhibitors had no effect, suggesting that the chemotactic response requires only PI3K activity (**Fig. 3F** **and** **4B**).

### Correlation between profilin-1 levels and atherosclerosis *in vivo*

**Figure 5:**
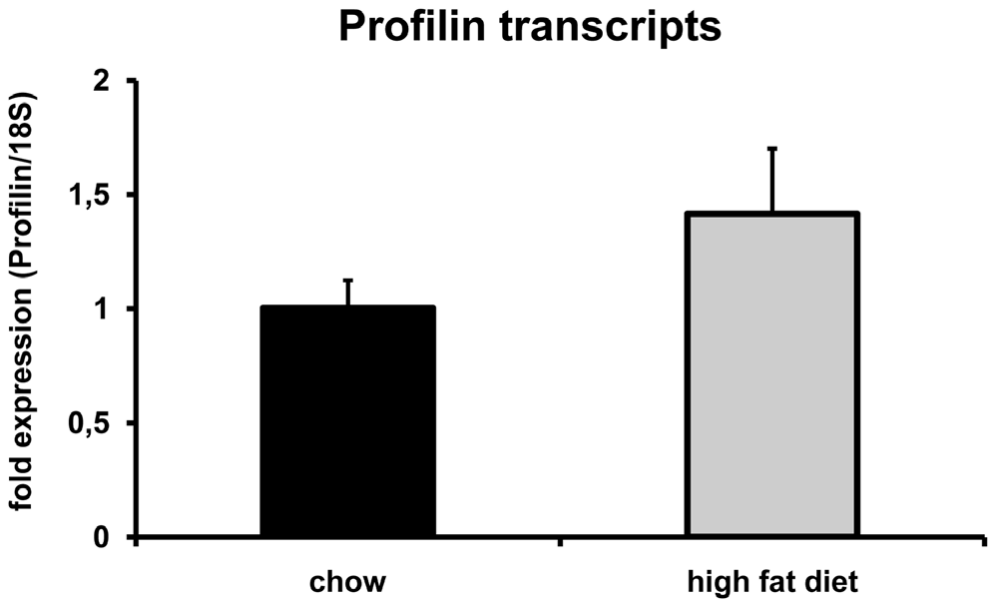
Profilin transcript levels in carotid arteries from normal or atherogenic LDLR-deficient mice. 6-month-old male LDLR-deficient mice were exposed to chow (n = 4) or atherogenic (n = 5) diet for 3 months. Subsequently, carotid arteries were removed, and profilin transcripts were measured by quantitative rt-PCR. Data are expressed as means ± SEM.

To assess whether pro-atherosclerotic stimuli affect the expression of profilin-1 in the vascular wall, the impact of atherogenic diet on transcript levels of profilin-1 was investigated in LDL receptor-deficient mice that were fed either chow or high fat diet. As shown in **Figure 5**, there was a clear trend towards increased levels of profilin-1 expression in animals that received high fat diet and subsequently developed atherosclerosis. Although this difference did not reach statistical significance, our data suggest that there might be a link between pro-atherogenic stimuli and vascular profilin-1 expression.

**Figure 6:**
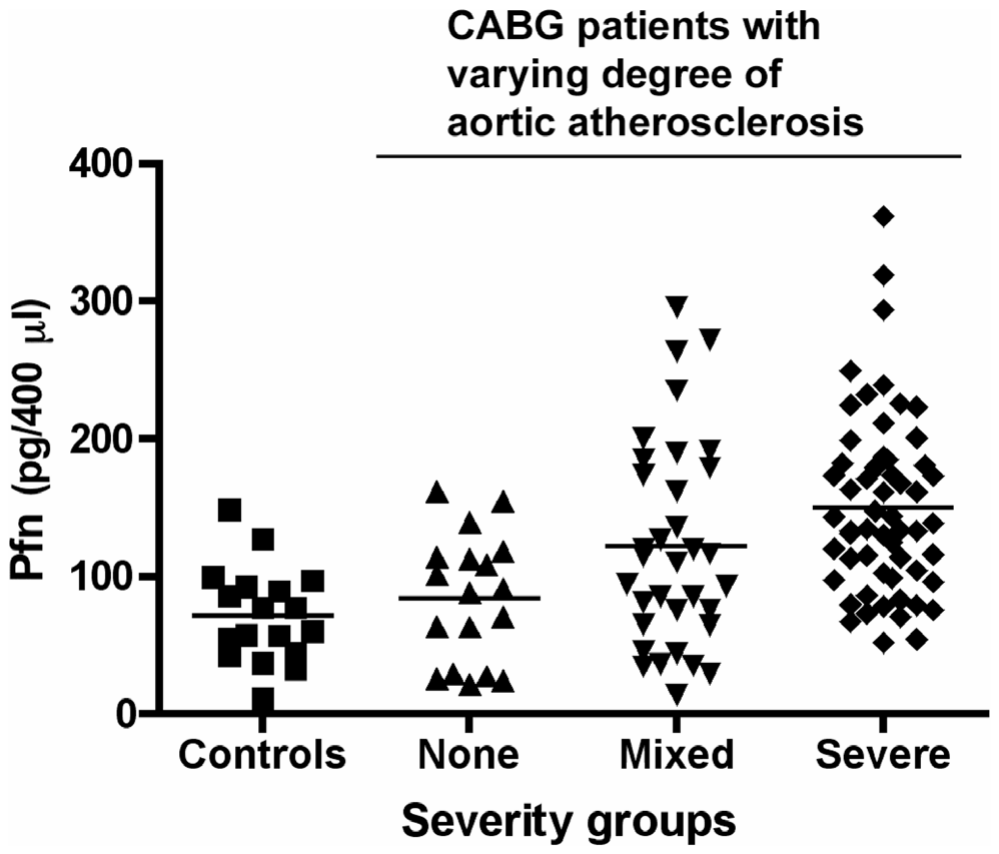
Correlation between the degree of aortic atherosclerosis and profilin-1 serum levels. In 104 patients undergoing CABG, the degree of aortic atherosclerosis was assessed by intraoperative ultrasound at 33 points along the arch, thoracic and abdominal aorta. According to the 33-point ultrasound score, individuals were clustered into three categories (0–7 = None; 8–20 = Mixed; 21–33 = Severe). The control group is represented by 18 individuals without major diagnosed diseases. Shown is a dot plot, the bar representing the mean in each group. *p*<0.001 between the ‘Severe’ group and the ‘Control’ or ‘None’ group.

Importantly, we also assessed whether the serum levels of profilin-1 were associated with the degree of atherosclerosis in humans. To this end, patients undergoing coronary bypass surgery were evaluated for the presence and severity of aortic atherosclerosis by intraoperative ultrasound at 33 points along the arch, thoracic and abdominal aorta, and the atherosclerosis score was correlated to profilin-1 serum levels. We found that profilin levels were significantly elevated in patients with the most severe aortic atherosclerosis score (**Figure 6**), indicating that profilin-1 serum levels were associated with the degree of atherosclerosis in humans.

## Discussion

The present study demonstrates that profilin-1 is highly expressed in human atherosclerotic plaques, and – although mainly recognized as an intracellular actin-binding protein – serves as an extracellular ligand and induces atherogenic effects in VSMCs such as DNA synthesis and migration. Furthermore, profilin-1 activates classical signaling pathways including the PI3K/Akt and Ras-Raf-MEK-Erk pathways. Profilin-induced chemotaxis of VSMCs is dependent on PI3K activity, whereas cell cycle progression requires multiple signaling pathways including PI3K, Src, and Erk1/2, but not PLCγ. Consistent with our previous studies, these data indicate that profilin-1 may be critically involved in the pathogenesis of atherosclerotic vascular disease.

Individuals with coronary risk factors including type 2 diabetes have a markedly elevated atherosclerotic risk [20]. The fact that the incidence of both diabetes and atherosclerotic vascular disease increases worldwide while diabetes-associated macrovascular disease is already a major health care issue, stresses the need for a better understanding of the pathomechanisms triggering atherosclerosis. Our previous work demonstrated that profilin-1 is present on the luminal surface of the endothelium in human and experimental diabetes, and that increased profilin-1 levels triggered various indicators of endothelial dysfunction [8]. In addition, LDL elevated profilin-1 levels in endothelial cells, which may be important as local accumulation of LDL in lipid-laden macrophages could further upregulate the expression of profilin-1 within plaques. Importantly, attenuation of profilin-1 expression leads to decreased atherosclerotic lesion formation in mice [9]. The present study indicates that profilin-1 expression is markedly elevated in human coronary plaques when compared to the normal vessel wall, and that profilin-1 expression is not restricted to the endothelium but is rather found throughout the plaque. Profilin-1 partly co-localized with VSMCs, but was also found in the extracellular space, as it may be released from ECs or apoptotic cells. Hence, in addition to affecting the integrity and function of endothelial cells, profilin-1 might also exert important paracrine effects within atherosclerotic lesions and thereby contribute to lesion progression.

The proliferation and migration of VSMCs are crucial events in the development and progression of atherosclerotic lesions [1], [4]. Quiescent VSMCs located in the media of undiseased arteries proliferate at a low frequency, and are arrested in the G(0)/G(1) phase of the cell cycle. In response to endothelial dysfunction or arterial injury, migration and subsequent proliferation of VSMCs occurs, particularly in patients with type 2 diabetes [1], [4], [21]. Although diabetes is associated with VSMC accumulation in developing lesions, hyperglycemia and hyperinsulinemia are unable to directly induce VSMC proliferation or migration *in vivo* [1]. Instead, risk factor-associated chronic inflammatory reactions and/or other mediators may be involved, and the classical risk factors (e.g., obesity, hypertension, dyslipidemia) are likely to act in concert with diabetes to promote the atherogenic process [2], [3]. Our data indicate that one such mediator may be profilin-1, which is known to be important for normal cell proliferation and differentiation. Its gene disruption leads to embryonic lethality due to grossly impaired growth, motility, and cytokinesis in single cells [22], [23], [24]. We show that profilin-1 acts as a potent mitogen and chemoattractant for VSMCs isolated from both rat aorta and human coronary arteries. These findings are consistent with previous studies demonstrating that profilin-1 is released into the extracellular space in pathological conditions such as experimental glomerulonephritis [14], and that profilin-1 exerts cellular responses like DNA synthesis and upregulation of AP-1 DNA-binding activity in mesangial cells via activation of cell surface receptors [15]. Furthermore, in line with our results transgenic overexpression of profilin-1 targeted to VSMCs caused vascular hypertrophy and hypertension in mice, and this was associated with significant increases in phospho-ERK1/2, phospho-JNK, and phospho-ROCK II kinase in mouse aortas [25].

While our data and other studies indicate that profilin-1 may act as an extracellular ligand, the mechanism of cellular activation remains elusive. One study suggested that a protozoan profilin-like protein activates a Toll-like receptor (TLR11) of dendritic cells [26]. However, a similar mechanism in rodents or humans has not been shown, so the role of Toll-like receptors in profilin signaling is questionable at present. Another study demonstrated that activation of DNA synthesis and AP-1 was associated with the binding of profilin-1 to cell surface receptors in rat mesangial cells [15]. Thus, although profilin may to act via a cell surface receptor, a specific receptor has not yet been identified and cloned, and little is known thus far about profilin signaling.

We have begun to identify the signaling pathways that mediate profilin-dependent cellular responses in VSMCs. Using specific pharmacological inhibitors, we found that profilin-dependent DNA synthesis required multiple signaling events including PI3K, Src, and Erk1/2 activity, whereas PLCγ was not required. These findings were not surprising, as PI3K, Src, and Erk1/2 were previously shown to be involved in cell proliferation in response to various stimuli [17], [27], [28]. Profilin-induced chemotaxis was attenuated only by inhibition of PI3K, whereas the presence of all other inhibitors did not affect the migratory response. This finding is also consistent with other reports demonstrating that PI3K signaling is critical for cellular motility and chemotaxis [17], [28].

Our further studies revealed that the vascular expression and serum levels of profilin-1 are associated with atherosclerosis *in vivo*. This was shown in LDLR-deficient mice in which the induction of atherosclerosis by high fat diet led at least to a trend of increased profilin-1 expression in the vessel wall, as found in human atherosclerotic plaques. These data are accompanied by the observation that patients with severe aortic atherosclerosis have significantly increased profilin-1 serum levels. Although we did not specifically correlate profilin levels with the severity of coronary artery disease for technical reasons, all patients in the analysis were admitted for CABG because of advanced coronary atherosclerosis, but the degree of atherosclerosis could be reliably quantified in the aorta. Nevertheless, the association between profilin-1 levels and coronary atherosclerosis should be analyzed in a larger cohort of patients in future studies.

In summary, we have demonstrated for the first time that profilin-1 is highly expressed in human atherosclerotic plaques, exerts direct atherogenic effects on VSMCs, and its serum levels are associated with the degree of atherosclerotic vascular disease in humans. Together with our previous reports, these data suggest that profilin-1 may play a role in early and advanced stages of atherosclerosis. Profilin-1 may therefore represent an important novel target for therapeutic and preventive strategies against atherosclerosis.
